# Possible Involvement of Hypothalamic Dysfunction in Long COVID Patients Characterized by Delayed Response to Gonadotropin-Releasing Hormone

**DOI:** 10.3390/ijms27020832

**Published:** 2026-01-14

**Authors:** Yuki Otsuka, Yoshiaki Soejima, Yasuhiro Nakano, Atsuhito Suyama, Ryosuke Takase, Kohei Oguni, Yohei Masuda, Daisuke Omura, Yasue Sakurada, Yui Matsuda, Toru Hasegawa, Hiroyuki Honda, Kazuki Tokumasu, Keigo Ueda, Fumio Otsuka

**Affiliations:** Department of General Medicine, Okayama University Graduate School of Medicine, Dentistry and Pharmaceutical Sciences, 2-5-1 Shikata-cho, Kita-ku, Okayama 700-8558, Japan; otsuka@s.okayama-u.ac.jp (Y.O.); yoshiakisoejima@s.okayama-u.ac.jp (Y.S.); y-nakano@okayama-u.ac.jp (Y.N.); asuyama@s.okayama-u.ac.jp (A.S.); p4v05asb@okayama-u.ac.jp (R.T.); oguni-gim@s.okayama-u.ac.jp (K.O.); yoheimasuda@s.okayama-u.ac.jp (Y.M.); me20011@s.okayama-u.ac.jp (D.O.); pzaf6h9w@s.okayama-u.ac.jp (Y.S.); phvw0350@okayama-u.ac.jp (Y.M.); pwcp0od9@s.okayama-u.ac.jp (T.H.); ppgf1hrd@okayama-u.ac.jp (H.H.); tokumasu@okayama-u.ac.jp (K.T.); p02n620b@okayama-u.ac.jp (K.U.)

**Keywords:** COVID-19, gonadotropin, gonadotropin-releasing hormone (GnRH), hypothalamus, long COVID

## Abstract

Long COVID (LC) may involve endocrine dysfunction; however, the underlying mechanism remains unclear. To examine hypothalamic–pituitary responses in patients with LC, we conducted a single-center retrospective study of patients with refractory LC referred to our University Hospital who underwent anterior pituitary stimulation tests. Between February 2021 and November 2025, 1251 patients with long COVID were evaluated, of whom 207 (19%) had relatively low random ACTH or cortisol levels. Ultimately, 16 underwent anterior pituitary stimulation tests and were included. All tests were performed in an inpatient setting without exogenous steroids. Fifteen patients (six women, mean age 35.6 years) underwent corticotropin-releasing hormone (CRH), thyrotropin-releasing hormone (TRH), and gonadotropin-releasing hormone (GnRH) tests. All patients had mild acute COVID-19, eight had ≥2 vaccinations, and the mean interval from infection was 343 days. Frequent symptoms included fatigue (100%), insomnia (66.7%), headache (60.0%), anorexia/nausea (40.0%), and brain fog (40.0%). Mean early-morning cortisol and 24 h urinary free cortisol were 7.5 μg/dL and 41.0 μg/day, respectively. MRI showed an empty sella in one case. Peak hormonal responses were preserved (ΔACTH 247%, ΔTSH 918%, ΔPRL 820%, ΔFSH 187%, ΔLH 1150%); however, peaks were delayed beyond 60 min in ACTH (13%), LH (33%), and FSH (87%). Notably, significantly delayed elevations remained at 120 min in the responses of TSH (4.1-fold), PRL (1.8-fold), LH (9.3-fold), and FSH (2.8-fold), suggesting possible hypothalamic involvement, particularly in the gonadotropin responses. Additionally, serum IGF-I was lowered (−0.70 SD), while GH response (mean peak 35.5 ng/mL) was preserved by growth hormone-releasing peptide (GHRP)-2 stimulation. Low-dose hydrocortisone and testosterone were initiated for three patients. Although direct viral effects and secondary suppression have been proposed, our findings may suggest that, at least in part, the observed response characteristics are consistent with functional secondary hypothalamic dysfunction rather than irreversible primary injury. These findings highlight the need for objective endocrine evaluation before initiating hormone replacements.

## 1. Introduction

Post-acute coronavirus disease 2019 (COVID-19) syndrome (long COVID) is characterized by persistent symptoms, such as fatigue, sleep disturbance, headache, and brain fog, which last for more than two to three months after recovery from acute infection [1]. Approximately one-third of patients with acute COVID-19 experience some form of long-term symptoms, although the prevalence estimates vary across studies [2,3]. Its pathophysiology remains unclear and is believed to involve multiple interacting factors, including immune, autonomic, vascular, metabolic, and mitochondrial dysfunction [4,5,6,7]. Among these mechanisms, growing attention has focused on abnormalities in the neuroendocrine system, particularly the hypothalamic–pituitary–adrenal (HPA) axis [8,9,10,11,12].

Severe acute respiratory syndrome coronavirus 2 (SARS-CoV-2) can infect hypothalamic and pituitary cells through ACE2 and TMPRSS2 receptors, and autopsy studies have demonstrated the presence of the virus in these regions [13]. Poma et al. reported SARS-CoV-2 antigens in the pituitary glands of patients who died from COVID-19, suggesting viral tropism for the human pituitary and encouraging the exploration of pituitary dysfunction following COVID-19 [14]. Notably, the transcripts of pituitary hormones and developmental or regulatory genes were suppressed in all COVID-19 cases, irrespective of the presence of the virus. Moreover, viral infection activates interferon responses and neutrophil and cytotoxic pathways, leading to reduced transcription of pituitary-specific hormones regardless of direct viral invasion to pituitary cells [14]. Inflammatory cytokines such as interleukins and tumor necrosis factors affect the hypothalamic and pituitary interaction and its neuronal activity, and central stress response dysfunction may persist into the chronic phase [15,16].

On the other hand, fatigue is the most common symptom of long COVID, and in clinical practice, some patients receive empirical steroid supplementation under the assumption of adrenal insufficiency [17]. However, prolonged post-COVID hypopituitarism lasting more than one year and cases with empty sella have been reported in Japan and abroad [18], suggesting that structural or functional impairment of the hypothalamus or pituitary can occur in a subset of patients. To date, most studies have relied on basal hormone testing, which is insufficient for detecting subtle hypothalamic dysfunction. Since establishing our specialized clinic for long COVID in 2021, we have experienced over 1200 patients with long COVID, and in several cases with marked fatigue, we suspected secondary hypothalamic dysfunction during clinical evaluation [19].

These observations prompted us to consider that a comprehensive assessment using dynamic anterior pituitary stimulation tests may be necessary. Therefore, the present study aimed to clarify hypothalamic–pituitary response patterns in long COVID by analyzing the results of these endocrine tests.

## 2. Results

**Figure 1:**
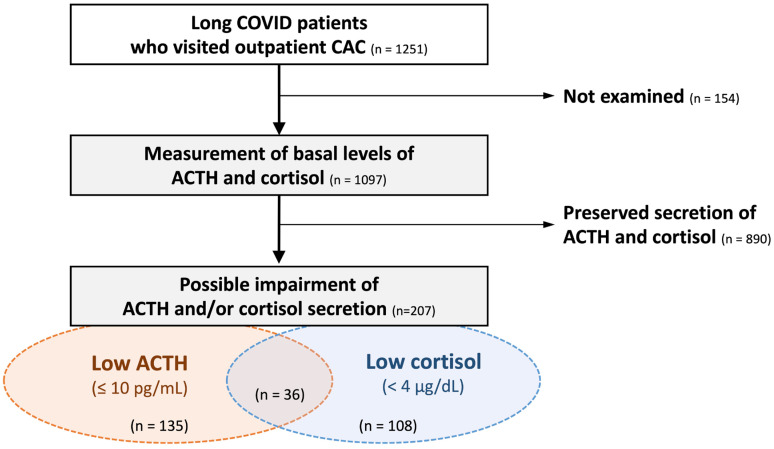
Screening and the prevalence of hypothalamic–pituitary–adrenal axis hypofunction among long COVID patients. Among a total of 1251 patients with long COVID, basal ACTH and cortisol levels were measured in 1097 patients. Decreased random ACTH and cortisol levels were observed in 135 (12.3%) and 108 (9.8%) patients, respectively. Either abnormality was present in 207 patients (18.9%), representing the screening-based prevalence of possible adrenal insufficiency after COVID-19 infection. CAC: COVID-19 aftercare clinic.

During the study period, 1251 patients with long COVID were eligible for preliminary screening. Random ACTH and/or cortisol levels were measured in 1097 patients based on clinical necessity. As a result, low random ACTH levels (≤10 pg/mL) were observed in 135 patients (12.3%), low random cortisol levels (<4 μg/dL) in 108 patients (9.8%), either finding in 207 patients (18.9%), and both findings in 36 patients (3.3%; Figure 1).

Ultimately, 16 patients met the inclusion criteria of main study, and 15 (9 men and 6 women; mean age, 35.6 years) underwent the complete set of corticotropin-releasing hormone (CRH), thyrotropin-releasing hormone (TRH), and gonadotropin-releasing hormone (GnRH) tests. All had experienced mild acute COVID-19, eight patients had received two or more vaccinations, and the mean interval from infection to evaluation was 343 days (Table 1). Comorbidities included dyslipidemia, hypertension, asthma, and Behçet disease (one each). Five patients (33.3%) were receiving selective serotonin reuptake inhibitors or dopamine agonists. One patient was taking 5 mg prednisolone, two used inhaled or intranasal steroids, and two were receiving hydrocortisone (10 and 15 mg daily) for clinically presumed adrenal insufficiency after COVID-19.

The major chief complaints among the 15 patients were fatigue (15 patients, 100%), insomnia (10 patients, 66.7%), headache (9 patients, 60.0%), anorexia/nausea (6 patients, 40.0%), and brain fog (6 patients, 40.0%; Table 1). Additional minor symptoms included fever, dizziness, dyspnea, abdominal pain, cold intolerance, weight loss, muscle weakness, tinnitus, sense of powerlessness, chest discomfort, stiffness, syncope, diarrhea, bowel habit changes, olfactory disturbance, chest pain, depressed mood, arthralgia, dry mouth, palpitations, and sore throat.

The baseline endocrine data for the 15 patients are shown in Table 2. Morning cortisol levels and 24-h urinary free cortisol levels averaged 7.5 µg/dL and 41.0 µg/day, respectively, suggesting low likelihood of adrenal insufficiency. Serum prolactin (PRL) levels were elevated above baseline in seven patients (46.7%), suggesting the possibility of hypothalamic impairment, but other pituitary hormones were within the normal ranges. Serum insulin-like growth factor (IGF)-I levels showed a slight, non-significant decrease, with a mean value of −0.8 SD (Table 2).

**Figure 2:**
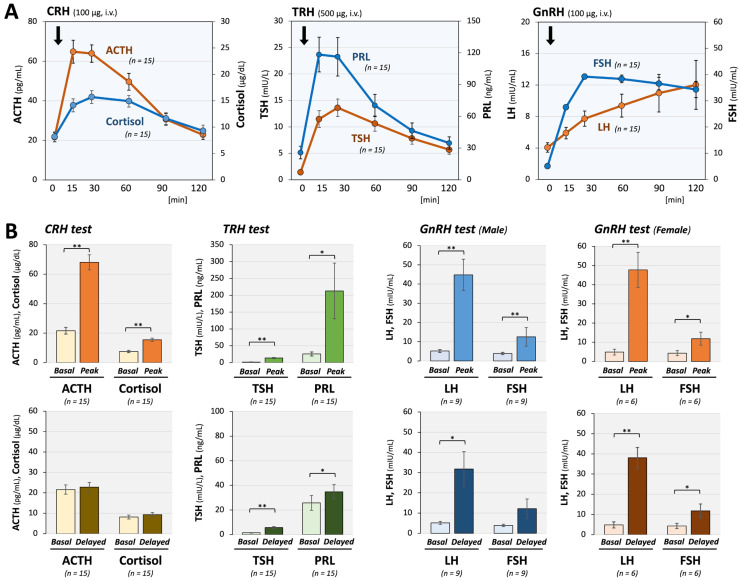
Pituitary responses to CRH, TRH, and GnRH stimulation in long COVID patients. (**A**) Time-dependent changes in pituitary hormones are shown. In 15 long COVID patients, anterior pituitary responses to stimulation with CRH (100 μg), TRH (500 μg), and GnRH (100 μg) were largely maintained; however, significant delays in peak responses, particularly for LH and FSH, were observed. These delay patterns suggest hypothalamic dysfunction rather than primary pituitary dysfunction. (**B**) Comparison of mean basal and peak hormone levels showed significant and substantial increases in all anterior pituitary hormones, supporting the conclusion that pituitary dysfunction is unlikely. In contrast, 120 min after stimulation, TSH, PRL, male LH, female LH, and female FSH significantly increased from baseline (1.4 vs. 5.7 mIU/L, 25.7 vs. 34.8 ng/mL, 5.2 vs. 31.8 mIU/mL, 4.9 vs. 38.1 mIU/mL, and 4.3 vs. 11.8 mIU/mL, respectively), demonstrating a prolonged response suggestive of hypothalamic dysfunction. The arrows indicate the time points of hormone injection. * *p* < 0.05 and ** *p* < 0.01 indicate significant differences between the indicated groups.

Pituitary magnetic resonance imaging (MRI) revealed an empty sella in one case; however, no other structural abnormalities were identified in the remaining 14 cases. The results of CRH, TRH, and GnRH tests revealed preserved peak responses (mean increases: Δadrenocorticotropin (ACTH) 247%, Δthyrotropin (TSH) 918%, ΔPRL 820%, Δfollicle stimulating hormone (FSH) 187%, Δluteinizing hormone (LH) 1150%); however, the peak hormone concentrations were observed after 60 min in ACTH, LH, and FSH among 2 cases (13.3%), 5 cases (33.3%), and 13 cases (86.6%), respectively (Figure 2A). Notably, significant sustainment of pituitary hormones after stimulation was detected at 120 min for TSH (4.1-fold), PRL (1.8-fold), LH (9.3-fold), and FSH (2.8-fold), compared with baseline levels (Figure 2B). After excluding patients with prior systemic glucocorticoid exposure, delayed and sustained responses remained evident. These changes indicated a particularly delayed and hyper-responses of FSH and LH secretions to the exogenous GnRH stimulation, suggesting impaired function of the GnRH-to-gonadotrope axis even in hypothalamic dysfunction.

**Figure 3:**
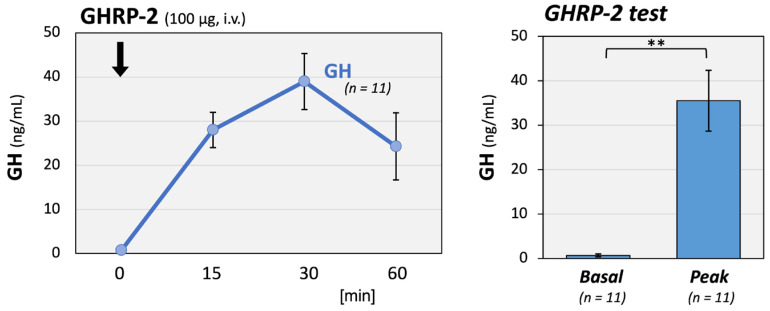
GH responses to GHRP-2 stimulation in long COVID patients. GH responses to GHRP-2 (100 μg) stimulation were well maintained in 11 long COVID patients. The left panel shows the time course of serum GH concentrations, and the right panel shows the basal and peak GH levels after stimulation. The mean GH peak level reached 35.5 ng/mL, a significant increase from baseline. The arrow indicates the time point of hormone injection. ** *p* < 0.01 indicates a significant difference between the indicated groups.

Additionally, growth hormone-releasing peptide (GHRP)-2 testing was conducted on 11 patients to detect growth hormone (GH) deficiency among total 16 patients. The means of their serum GH and insulin-like growth factor-I (IGF-I) of these 11 patients were 0.36 ng/mL and −0.70 SD, respectively. GH responses were preserved with a significantly elevated mean peak of 35.5 ng/mL (Figure 3).

Of the two patients who had already started receiving steroid replacement before visiting our institution, tapering was possible in both cases. In addition, a small dose of hydrocortisone was newly initiated in two patients and androgen supplementation in one, among the 16 patients included in this study.

## 3. Discussion

In the present study, patients with long COVID who underwent multiple anterior pituitary stimulation tests were considered to have possible hypothalamic–pituitary involvement. Although the peak responses were preserved across the axes, we observed consistent patterns of delayed peaks and sustained elevation at 120 min, especially in the responses of gonadotropins, PRL, and TSH. These findings are consistent with, but not specific to, a well-recognized pattern of hypothalamic dysfunction, in which the pulsatile release of hypothalamic hormones is impaired while pituitary secretory capacity remains intact [20,21]. The clear peak responses indicate that pituitary function itself was preserved. Delayed and prolonged responses indicate a reduction in hypothalamic hormonal drive. Our current study, especially the characteristic gonadotrope responsiveness to GnRH, suggested a possible hypothalamic impairment of GnRH secretion in long COVID patients.

The marked delay and sustained responses of LH and FSH may be related to intrinsic features of the hypothalamic–pituitary–gonadal (HPG) axis. Gonadotropin secretion depends on precise GnRH pulsatility, gonadal feedback, and high sensitivity of pituitary gonadotrophs. Basic experimental studies have demonstrated that GnRH and CRH bind rapidly to pituitary membranes, undergo prompt internalization, and are generally processed within approximately 30 min [22]. Because stimulation tests bypass endogenous hypothalamic hormone release, these findings do not allow direct assessment of hypothalamic secretory dynamics. Nevertheless, a delayed peak is unlikely to arise from intrinsic pituitary mechanisms and instead suggests impaired hypothalamic input, specifically insufficient pulsatile drive or an inability to terminate upstream stimulation appropriately. LH and FSH responses are particularly susceptible to alterations in hypothalamic input [23]. These characteristics allow gonadotropins to serve as more sensitive indicators of subtle hypothalamic dysfunction than other anterior pituitary hormones.

Several factors contribute to secondary hypothalamic dysfunction, including residual inflammation, autonomic dysregulation, and microcirculatory disturbances [11,24,25]. MRI in our cohort showed no significant structural abnormalities except for one case of an empty sella, and the GH–IGF axis remained preserved, suggesting functional rather than irreversible damage. Although GH responsiveness was intact, IGF-I standard deviation (SD) scores were modestly reduced to approximately “−1” SD. This pattern may reflect an altered nutritional or metabolic status, including increased catabolism or impaired carbohydrate balance, which could suppress IGF-I production. We previously reported that blood albumin levels and BMI are affected by the GH-IGF-I axis in patients with non-GH-related diseases [26]. Specifically, there was an enhanced negative correlation between GH and IGF-I under lean and low-nutrient conditions. Similarly, the mild reduction in IGF-I observed in our patients may be attributable to metabolic disturbances associated with long COVID.

Urhan et al. reported altered endocrine responses in patients after COVID-19, including insufficient cortisol responses to a low-dose ACTH test in 16.2% of cases and inadequate GH or cortisol responses to the glucagon stimulation test (GST) in 46.5% and 9.3% of cases, respectively [27]. Serum IGF-I levels were reduced in 9.3% of patients with impaired GH responses to GST. Mild TSH or PRL elevation and central hypogonadism were also observed in 9.3%, 4.6%, and 9.3% of the patients, respectively [27]. These findings suggest that COVID-19 can affect the pituitary function, particularly the HPA and GH axes. However, the discrepancies between their findings and ours may reflect differences in various factors, including the disease phases (acute vs. long COVID), viral variants [28], and vaccination status. In our preliminary study, we observed that approximately 19% of patients, including asymptomatic cases, had relatively low randomized ACTH and/or cortisol levels. This is roughly consistent with the prevalence of low cortisol levels observed in a previous report on COVID-19 patients who did not exhibit specific symptoms due to infection [29]. The number of patients undergoing stimulation tests may have been underestimated due to clinical relevance and feasibility.

Given that our patients with long COVID had been symptomatic for more than 300 days on average [30], the long-term illness burden and associated physiological stress may also have contributed to hypothalamic impairment. Although aging is associated with reduced GnRH secretion, the predominant abnormality is decreased pituitary functions, and delayed or sustained hyper-responses are not characteristic. Obesity also influences hypothalamic and pituitary functions; however, primary alterations occur in the GH axis rather than in the HPG axis. These differences suggest that the delayed and prolonged gonadotropin responses observed in long COVID patients are due to mechanisms distinct from the reported aging- and metabolism-related changes [24,31].

The hypothalamus regulates not only pituitary secretion but also sleep–wake rhythms, thermoregulation, autonomic tone, and emotional responses [32]. Considering these functions, hypothalamic dysfunction provides a plausible explanation for various long COVID symptoms [33], including sleep disturbances, functional hyperthermia, postural intolerance, and affective changes, and may be relevant to brain fog [34]. Studies comparing Sjögren syndrome, which causes fatigue and sleep disturbances similar to long COVID, to fibromyalgia have shown that exaggerated pituitary responses on stimulation testing may reflect underlying hypothalamic dysfunction [35].

We previously reported that men with long COVID not rarely experience late-onset hypogonadism (LOH) syndrome, a decrease in testosterone associated with andropause [19,25,36]. Meanwhile, approximately 20% of women with long COVID experience menstrual abnormalities, particularly menstrual cycle abnormalities, which can lead to depressive mood [37]. Both male LOH and female menstrual abnormalities may be due to primary hypogonadism as well as secondary hypogonadism caused by hypothalamic and pituitary dysfunction, making differentiation difficult [38]. Our present results of the pituitary stimulation testing performed in long COVID patients suggest that hypothalamic dysfunction, including gonadotropes, contributes, at least in part, to the hypogonadism observed in both men and women with long COVID.

However, in the present patients with long COVID, the peak ACTH and cortisol responses to CRH and their recovery levels at 120 min were nearly normal, failing to detect clear HPA axis abnormalities. Furthermore, assessment of basal cortisol secretion using 24 h urinary collection (UFC) revealed a nearly normal level of approximately 40 μg/day. Normally, a hypoglycemic challenge test using insulin is necessary to clarify hypothalamic dysfunction; however, because of the significant fatigue, shortness of breath, and mental and physical instability experienced by patients with long COVID, this test was not performed to avoid the risk of hypoglycemia. An insulin challenge test is preferred for a more accurate assessment of hypothalamic function.

Autoimmune/inflammatory syndrome induced by adjuvants, an exaggerated immune response observed after COVID-19 infection or vaccination, has been discussed in relation to long COVID or ME/CFS. COVID-19 infection has been reported to induce anti-pituitary and anti-hypothalamic antibodies [39], leading to autoimmune hypophysitis or hypothalamitis, raising the possibility that such immune mechanisms underlie the hypothalamic dysfunction observed in our study. However, data on GnRH-related responses during the chronic phase of COVID-19 remain limited, and further research is needed to clarify the underlying mechanisms.

Although glucocorticoid replacement is appropriate for confirmed adrenal insufficiency, excessive or empiric steroid use may suppress hypothalamic and pituitary functions. Our findings raise the concern that indiscriminate steroid administration in long COVID could worsen hypothalamic suppression and prolong dependency on steroids. When the symptoms are severe, temporary low-dose hormone replacement therapy may be justified [40]; however, careful monitoring and gradual tapering are essential. In contrast, abnormal gonadotropin responsiveness was observed in men and women, suggesting hypothalamic menopausal pathology in both sexes. This finding highlights the need to evaluate the steroid secretory status and functional attenuation of the HPG axis in future patients with long COVID.

This study has several limitations. First, owing to its retrospective design, analyses incorporating appropriate control groups were not feasible, which limits causal inference. Second, anterior pituitary stimulation testing was performed strictly based on clinical necessity, and therefore, sampling bias toward patients with more prominent or refractory neuroendocrine-like symptoms cannot be excluded. Third, the small sample size reflects the exploratory and hypothesis-generating nature of this study and limits statistical power. Fourth, although analyses excluding patients with prior systemic glucocorticoid exposure were performed, the potential influence of concomitant medications, including steroids and other drugs affecting pituitary hormone secretion, could not be completely eliminated. Fifth, hypothalamic function could not be directly assessed using insulin-induced hypoglycemia or other definitive tests because of safety concerns in patients with long COVID. Therefore, the present findings should be interpreted as hypothesis-generating rather than confirmatory. Finally, although elevated prolactin levels were observed in some patients, detailed evaluation for macroprolactinemia was not performed, and its contribution cannot be ruled out.

## 4. Materials and Methods

### 4.1. Preliminary Screening

As a preliminary screening step, we first identified patients aged 10 years or older who visited the Department of General Medicine at Okayama University Hospital, a Japanese tertiary hospital, between February 2021 and November 2025 for persistent symptoms following COVID-19. Among these patients, those with suspected hypothalamic–pituitary–adrenal axis hypofunction were identified based on the laboratory result of random ACTH levels ≤ 10 pg/mL and/or serum cortisol levels < 4 μg/dL (based on the Japanese guideline at https://doi.org/10.1507/endocrine.99.S.July_1, accessed on 5 January 2026).

### 4.2. Study Design and Patient Selection

Subsequently, a single-center, retrospective, observational study was conducted using electronic medical records. The analysis included patients aged 10 years or older with confirmed or suspected long COVID who underwent anterior pituitary stimulation tests in the Department of General Medicine at Okayama University Hospital between February 2021 and November 2025 based on clinical indication for suspected endocrine dysfunction. No exclusion criteria were applied.

### 4.3. Basal Endocrine Data

From medical records, we obtained blood and urinary hormone levels. Blood samples were collected in the early morning under fasting and resting conditions to determine the basal levels of the following hormones: ACTH, cortisol, TSH, free thyroxin (FT4), PRL, LH, FSH, GH, and IGF-I. In detail, serum hormone concentrations were determined using the auto-analyzer system Cobas 8000 (F. Hoffmann-La Roche AG, Basel, Switzerland) at the Central Laboratory of Okayama University Hospital [11]. Serum FT4 and TSH were determined by Elecsys FT4 III and TSH kits (F. Hoffmann-La Roche AG), respectively, and serum cortisol and plasma ACTH were determined by Elecsys Cortisol II and ACTH kits (F. Hoffmann-La Roche AG), respectively [11]. Accordingly, serum LH and FSH were determined using Elecsys LH and FSH II kits (F. Hoffmann-La Roche AG), respectively. Serum GH and IGF-I were also examined using Elecsys GH and Elecsys IGF-I kits (F. Hoffmann-La Roche AG), respectively, and the IGF-I levels were evaluated using the SD values [41].

### 4.4. Endocrine Stimulation Tests

From medical records, we also obtained pituitary MRI findings and the results of CRH, TRH, GnRH (luteinizing hormone-releasing hormone: LHRH), and GHRP-2 stimulation tests. Anterior pituitary stimulation tests were performed in patients who initially underwent basal laboratory testing during outpatient evaluation for persistent post-COVID symptoms suggestive of possible neuroendocrine involvement, such as fatigue. Based on a comprehensive assessment integrating clinical symptoms and basal endocrine data, including relatively low random ACTH and/or cortisol levels, the indication for pituitary stimulation testing was discussed and confirmed by at least three board-certified endocrinologists. Notably, no predefined cutoff values for random ACTH or cortisol levels were used in this decision-making process; rather, eligibility was determined through an overall clinical judgment. In addition, patient preference and practical feasibility were considered, and therefore, stimulation tests may not have been performed in all patients who were considered potentially eligible. In patients who received oral steroids, the dose was withheld on the test day. Blood samples for each hormone were obtained at baseline (pre-stimulation) and at 15, 30, 60, 90, and 120 min following administration of the respective releasing hormones, except for the GHRP-2 test, in which samples were collected at baseline and at 15, 30, and 60 min. Basal values, peak values, time to peak, and 120 min values were evaluated, and the response ratios were calculated by dividing the measured values by basal values. In accordance with previous reports, peak responses observed beyond 60 min were considered delayed [20,21].

### 4.5. Assessment of COVID-19

We also extracted background information, symptoms, past medical history, medication, severity of acute COVID-19, interval from infection to our evaluation, and COVID-19 vaccination history. The diagnosis and severity of acute COVID-19 were based on referral letters, medical records from referring physicians, and patient reports; serological antibody testing was used when necessary to confirm consistency. Long COVID was diagnosed by each treating physician according to established criteria [42].

### 4.6. Statistical Analysis

All analyses were conducted using EZR, version 1.68 (Saitama Medical Center, Jichi Medical University, Saitama, Japan), which is a graphical user interface for R, version 4.3.1 (The R Foundation for Statistical Computing, Vienna, Austria) [43]. Continuous variables are expressed as mean and standard errors (SEs). Paired *t*-tests were used to assess changes in hormone levels, with *p* < 0.01 or 0.05 considered statistically significant. Missing data were excluded from the analysis.

### 4.7. Ethical Considerations

This study was approved by the Institutional Review Board of Okayama University Hospital (No. 2601-042 and No. 2105-030) and performed in accordance with the Declaration of Helsinki. The requirement for individual consent was waived owing to the retrospective design, and the information was disclosed to the participants through an opt-out consent process.

## 5. Conclusions

In conclusion, this study provides comprehensive data from dynamic endocrine testing in patients with long COVID, demonstrating a pattern consistent with functional hypothalamic dysfunction, particularly in hypothalamic gonadotropin responsiveness. Further research integrating endocrinology, infectious disease, and immunology perspectives is needed to elucidate the effects of SARS-CoV-2 on the neuroendocrine system and develop appropriate therapeutic strategies.

## Figures and Tables

**Table 1:** Background characteristics of long COVID patients who underwent pituitary stimulation tests.

Background of Long COVID Patients	Number (%)
Sex (*n*, %)	
Female/male	6 (40.0%)/9 (60.0%)
Age (years, mean ± SE)	35.6 ± 3.8
BMI (kg/m^2^, mean ± SE)	25.7 ± 1.8
Interval from infection (days, mean ± SE)	343 ± 87
Acute COVID-19 Severity (*n*, %)	
Severe	0 (0%)
Moderate	0 (0%)
Mild	15 (100%)
Hospitalization status in acute phase (*n*, %)	
Hospitalized	0 (0%)
Home care	15 (100%)
Smoking habit (*n*, %)	1 (6.7%)
Drinking habit (*n*, %)	0 (0%)
COVID-19 vaccination (*n*, %)	
0–1 time	6 (40.0%)
More than 2 times	8 (53.3%)
No information	1 (6.7%)
**Chief complaints related to long COVID**	
Fatigue	15 (100%)
Insomnia	10 (66.7%)
Headache	9 (60.0%)
Loss of appetite and nausea	6 (40.0%)
Brain fog	6 (40.0%)
Cough	4 (26.7%)
Memory loss	4 (26.7%)
Fever	4 (26.7%)
Dizziness	4 (26.7%)
Dyspnea	3 (16.2%)

SE: standard error.

**Table 2:** Basal hormone levels of long COVID patients who underwent pituitary stimulation tests.

	Mean ± SE (*n* = 15)	Reference Range
ACTH (pg/mL)	22.3 ± 2.3	7.2–63.3
Cortisol (μg/dL)	7.5 ± 0.8	7.1–19.6
TSH (mIU/L)	1.4 ± 0.3	0.61–4.23
FT4 (ng/dL)	1.3 ± 0.0	0.97–1.69
PRL (ng/mL)	29.2 ± 9.7 (male) 20.5 ± 3.9 (female)	4.3–13.7 (male) 4.9–29.3 (female) ^†^
LH (mIU/mL)	5.2 ± 0.8 (male) 5.3 ± 1.8 (female)	2.2–8.4 (male) 1.4–15 (female) ^†^
FSH (mIU/mL)	3.9 ± 0.6 (male) 4.8 ± 1.8 (female)	1.8–12 (male) 3–10 (female) ^†^
GH (ng/mL)	0.52 ± 0.25 (male) 0.6 ± 0.32 (female)	<2.47 (male) 0.13–9.88 (female)
IGF-I (ng/mL)	181.4 ± 29.8	not applicable
IGF-I (SD)	−0.8 ± 0.4	−2.0–2.0

SE: standard error, SD: standard deviation. ^†^ Female reference ranges for PRL, LH, and FSH represent values in the follicular phase of premenopausal women.

## Data Availability

The data presented in this study are available on request from the corresponding author because the data are not publicly available due to privacy and ethical restrictions.

## Abbreviations

Coronavirus disease 2019 (COVID-19), corticotropin-releasing hormone (CRH), thyrotropin-releasing hormone (TRH), gonadotropin-releasing hormone (GnRH), growth hormone-releasing peptide (GHRP), hypothalamic–pituitary–adrenal (HPA), hypothalamic–pituitary–gonadal (HPG), insulin-like growth factor-I (IGF-I), and severe acute respiratory syndrome coronavirus 2 (SARS-CoV-2).
